# Hospital Meetings

**Published:** 1901-11-09

**Authors:** 


### HOSPITAL MEETINGS.

### EVELINA HOSPITAL FOR SICK CHILDREN.

A meeting of the Court of Governors of the Evelina Hospital for Sick Children was held on Wednesday, 30th ult., at the hospital. The following were present:—Messrs. J. Howard Colls, W. W. Palmer, Chas. D. Seligman, and W. Aste (in the chair). The Chairman said that the beds were very fully occupied; that from a financial point of view the hospital had managed to struggle along, and that this was all that could be said. Fresh subscribers were badly wanted. There were many gentlemen in the immediate neighbourhood whose names he thought it ought to be possible to add to the list; at present no support came from them. The Hon. Conrad Dillon had been in bad health for some time past, and he regretted to state that from the latest accounts there was no improvement, and that there was very little prospect of his being able to carry on his work as chairman of the committee of management; this was a cause of great grief to the committee. As they had only that day heard of his retirement, no successor had yet been elected. He might mention that Mr. Rothschild had visited the hospital a few days previously; he had remarked that the accommodation for the nurses and household staff was not what he would like to see; this opened up a very large question. The committee were giving it their consideration, and had called in a gentleman experienced in such matters; they expected soon to bring forward some scheme, and that those who had always helped the hospital would rally round and give what assistance they could. It was true that the accommodation referred to was not what it should be, and as they did not want to be behind the times he hoped the necessary means would not be lacking; he would like to urge upon those in the neighbourhood the necessity of helping. It was sometimes asked why no grant was received from the Prince of Wales's Fund; to this question the answer was that the money was only devoted to hospitals in which the wards had had to be closed for want of funds, and this had not been the case with the Evelina Hospital. He hoped next time the Court met there would be more to say about the alterations. It was proposed to place the Hon. Conrad Dillon's name on the books as vice-president, and to make Mr. F. W. Reynolds vice-chairman. There being no further business the meeting terminated with a vote of thanks to Mr. Aste for acting as chairman *pro tem.*

